# Hospital-wide education committees and high-quality residency training: A qualitative study

**DOI:** 10.1007/s40037-017-0390-9

**Published:** 2017-12-11

**Authors:** Milou E. W. M. Silkens, Irene A. Slootweg, Albert J. J. A. Scherpbier, Maas Jan Heineman, Kiki M. J. M. H. Lombarts

**Affiliations:** 1 Professional Performance Research group, Department for Educational Support, Academic Medical Center/University of Amsterdam, Amsterdam, The Netherlands; 2 Department of Educational Development and Research, Maastricht University, Maastricht, The Netherlands

**Keywords:** Postgraduate medical education, Residency training, Educational governance, Hospital-wide education committees

## Abstract

**Introduction:**

High-quality residency training is of utmost importance for residents to become competent medical specialists. Hospital-wide education committees have been adopted by several healthcare systems to govern postgraduate medical education and to support continuous quality improvement of residency training. To understand the functioning and potential of such committees, this study examined the mechanisms through which hospital-wide education committees strive to enable continuous quality improvement in residency training.

**Methods:**

Focus group studies with a constructivist grounded theory approach were performed between April 2015 and August 2016. A purposeful sample of hospital-wide education committees led to seven focus groups.

**Results:**

Hospital-wide education committees strived to enable continuous quality improvement of residency training by the following mechanisms: creating an organization-wide quality culture, an organization-wide quality structure and by collaborating with external stakeholders. However, the committees were first and foremost eager to claim a strategic position within the organization they represent. All identified mechanisms were interdependent and ongoing.

**Discussion:**

From a governance perspective, the position of hospital-wide education committees in the Netherlands is uniquely contributing to the call for institutional accountability for the quality of residency training. When implementing hospital-wide education committees, shared responsibility of the committees and the departments that actually provide residency training should be addressed. Although committees vary in the strategies they use to impact continuous quality improvement of residency training, they increasingly have the ability to undertake supporting actions and are working step by step to contribute to high-quality postgraduate medical education.

## What this paper adds

The traditional specialty-specific organization of residency training lacks institutional accountability. Therefore, several healthcare systems have implemented hospital-wide education committees (HECs) to govern quality improvement of residency training at the organizational level. This paper explored the mechanisms through which HECs strive to enable continuous quality improvement in residency training and thereby added to current insights on the potential of institutional accountability. The main message for healthcare systems that implemented or wish to implement HECs is: HECs can contribute to the quality of residency training, but a strategic position from which they have the authority to intervene is crucial to their success.

## Introduction

High-quality postgraduate medical education (PGME) is of utmost importance for residents to become competent medical specialists [1]. During the worldwide modernization of medical education, methods for continuous quality improvement of residency training (CQI) (e. g. the Plan-Do-Check-Act cycle (PDCA cycle)) [2] have been introduced to assure and improve the quality of PGME. Although such CQI efforts have shown promising improvements in teaching performance and learning climate [3, 4], the traditional specialty-specific organization of residency training means CQI of PGME does not receive attention at the organizational level. Therefore, exchange of policies, facilities and best practices for CQI of PGME are not shared within a teaching hospital, leaving unfulfilled potential to improve PGME quality. For this reason, bodies such as the American Accreditation Council on Graduate Medical Education (ACGME), the British General Medical Council (GMC) and the Royal Dutch Medical Association are calling for a shift from solely specialty-specific responsibility towards centralized institutional accountability in which residency training is a shared responsibility of educators and the teaching hospital [5–9]. Insight into how institutional accountability for PGME takes form is absent, hindering clarity about its potential for the quality of residency training.

When it comes to patient care, the concept of institutional accountability is not new to the medical world. Clinical governance is a well-established vehicle through which health organizations as a whole are accountable for the quality of patient care [10]. Clinical governance aims to integrate fragmented CQI efforts for patient care into a centralized internal quality management system [11]. Similarly, PGME should entail a solid internal quality management system that facilitates educational CQI to benefit the quality of residency training. Healthcare systems such as in the United States and the Netherlands have contributed to centralized internal systems for PGME by requiring teaching hospitals to install hospital-wide education committees (HECs) for overseeing the quality of residency programs. These committees monitor residency training and support CQI efforts for PGME within the hospital.

A case study by Curry et al. (2008) reports positive effects for educational quality as a result of installing an HEC, showing HECs can guide changes in residency programs, closing of unsuccessful programs and development of new programs [12]. Despite these promising observations, comprehensive insight into mechanisms through which HECs may contribute to PGME is missing. This study addresses this knowledge gap by answering the following research question: what are the mechanisms through which hospital-wide education committees strive to enable continuous quality improvement in postgraduate medical education? In answering this question, we aim to gain a deeper understanding of the value of HECs for the quality of residency training.

## Methods

### Setting

In the Netherlands residency training is organized in eight geographical regions, all consisting of a coordinating academic hospital and regional affiliated teaching hospitals. Regional affiliated hospitals are either top clinical hospitals (providing PGME, scientific research and specialized care; a top clinical qualification can be achieved by hospitals fulfilling requirements set by the Organization of Top Clinical Hospitals) or general hospitals (providing PGME and patient care). Residents work in a clinical department and are trained and supervised by a team of clinical teachers jointly responsible for training. Each training program is headed by a program director appointed by the Royal Dutch Medical Association.

The Dutch College for Medical Specialties (the legislative body for PGME accreditation) officially launched the modernization program of PGME in 2003 [13]. Since 2011, the Directive of the Central College of Medical Specialists, which defines rules and regulations for (i) the curriculum of residency training, (ii) the registration of program directors and (iii) the training institute, mandates teaching hospitals to have an operational HEC responsible for monitoring and promoting the quality of residency training [6]. Without such an HEC, hospitals cannot be assigned the status of teaching hospital. The core tasks of HECs include the protection of individual residents, facilitating high-quality residency training for the collective group of residents in the teaching hospital, and guaranteeing that training programs have supportive learning climates [6]. The directive states that HEC members should represent all residency program directors, residents and the hospital board. The internal quality management performed by HECs is additional and supportive to the accreditation process performed every 5 years by external auditors.

### Study design

We performed a focus group study with a constructivist grounded theory approach, because of the explorative nature of the study. All researchers participated in the iterative development of the initial research question and the co-construction of meaning and knowledge during data collection and analysis in order to create an understanding of HEC functioning [14, 15]. We allowed for interactive discussions between participants to capture a wide array of feelings, attitudes and opinions [16, 17].

### Study population and data collection

We purposefully selected a variety of teaching hospitals, based on type of hospital (academic, top clinical and general teaching hospitals), size and geographical location, to capture a diversity and richness of data ([15]; Table 1). Selection was performed iteratively in which every new inclusion was driven by previous inclusions. For every selected teaching hospital, chairs of the corresponding HECs were approached from February 2014 until January 2016 by a member of the research team (KL) via email to participate with daily board members of the HEC in a focus group study. Upon interest, the main researcher (MS) provided extensive information about the goals and procedures of the research to the chairs by email. Chairs then disseminated this information to the participating HEC members.

**Table 1:** Type of hospital from which hospital-wide educational committees (HECs) originated and formal positions of participants

Type of hospital^a^ and geographical region^b^	Formal positions of participants (P)
HEC1: top clinical teaching hospital, region A	P 1: Coordinating staff for PGME
	P 2: Advisor to the HEC
	P 3: Chair of the HEC
	P 4: Educational supporting staff
	P 5: Formal educator at the department
HEC2: general teaching hospital, region B	P 6: Formal educator at the department
	P 7: Formal educator at the department
	P 8: Vice formal educator at the departments
	P 9: Advisor to the HEC
	P 10: Successive chair of the HEC
HEC3: top clinical teaching hospital, region C	P 11: Vice chair of the HEC
	P 12: Resident representative
	P 13: Educational supporting staff
	P 14: Hospital board member and formal educator at the department
HEC4: top clinical teaching hospital, region D	P 15: Chair of the HEC
	P 16: Formal educator at the department and successive chair of the HEC
	P 17: Coordinating staff for PGME and formal educator at the department
	P 18: Secretary staff
HEC5: academic teaching hospital, region B	P 19: Coordinating staff for PGME
	P 20: Resident representative
	P 21: Vice chair of the HEC
	P 22: Formal educator at the department
HEC6: general teaching hospital, region C	P 23: Educational supporting staff
	P 24: Coordinating staff for PGME
	P 25: Secretary staff
HEC7: top clinical teaching hospital, region E	P 26: Vice formal educator at the department
	P 27: Resident representative
	P 28: Formal educator at the department
	P 29: Chair of the HEC and formal educator at the department

^a^Academic hospital (coordinating PGME for affiliated hospitals); top clinical hospital (providing specialized clinical care, scientific research and PGME); general hospital (providing patient care and PGME)

^b^Covered a total of 5 out of 8 geographical regions

Based on extensive discussions within the research group, a discussion guide with five open-ended questions was designed to structure the focus groups. Two questions were intended to explore and define internal quality management systems and the role of the HEC within this process. Two questions investigated processes and factors underlying and evolving from internal quality management. One wrap-up question asked participants for suggestions for other relevant topics that could be discussed and whether they could formulate take-home messages (Table 2). A skilled moderator (IS) facilitated the discussion and an observer (either the first author (MS) or fellow researchers from the research group) captured nonverbal communication. All focus groups were audio-taped and transcribed verbatim.

**Table 2:** Focus group guide used during the study

Number:	End:	Transcription:
Date:	Moderator	Setting:
Start:	Observer	No. participants:
Five main questions	Topic list^a^	
What is internal quality management and what is the role of the HEC herein?	Definition: – Quality assurance (PDCA cycle, performance measurements, performance evaluation, questionnaires) – Quality improvement (action, innovation, consolidation) – Internal auditing	
	Internal quality management versus external quality management	
	Parties involved: – Hospital board – HEC – Educationalists/advisors/supporting staff – Departments/teaching teams/clinical teachers – Residents – External coaches/parties	
	Role of HEC: – Promote/stimulate/intervene/execute – Birds-eye view/monitoring – Policy making – Take responsibility/facilitate meetings	
How do you feel about internal quality management?	Positive: – Good/important/enthusiastic/activating – Added value (improves quality of residency training) – Supportive to external quality management	
	Negative: – Bad/takes time/necessity – Too much work/not useful – Hierarchical	
What are achievements of internal quality management and the HEC’s effort?	Levels of impact: – Level of teaching hospital (hospital board, finances, HEC) – Level of departments (leadership, teaching teams, clinical teachers) – Level of residents (more residents, better residents, satisfaction) – Level of the patient (change in care)	
	Achievements: – More awareness/attention/interest – Impact of HEC (meetings, content, collaborations, facilities, power, finance) – Education (better programs, development, innovations) – Patient care	
What is needed to make internal quality management work? What impairs?	Needed: – Culture (collective vision, everybody on board, representatives, exchange of best practices) – Systems (routines) – Communication/collaboration/support – HEC power (right to intervene, freedom, trust)	
	Impairing: – External pressure – Excess work – Compulsivity/coercion/pressure – Pressure to perform more patient care – Lack of finance/support – Content of some tools used in internal quality management	
Are there remaining topics that were not discussed? What is your take home message?	– Innovations – Hospital merges – Large scale projects (building, expanding hospital) – Accreditation	

^a^The topic list was used only after initial discussion of the main question. In line with the iterative approach adopted in the study, the topic list was adjusted (mainly extended) for each new focus group, based on topics addressed in previous focus groups. The topic list was used to introduce points for discussion that were not mentioned during initial discussion (to broaden the focus group discussion)

A focus group consisted of members of an HEC within one hospital only. Focus groups were held between April 2015 and August 2016 and lasted a maximum of 75 min. Overall, seven focus group discussions with a total of 29 participants were conducted. No new codes emerged after the sixth focus group, confirming data saturation at the seventh focus groups. The number of participants per focus group and their formal positions varied (Table 1).

### Data analysis

Data collection, analysis (coding) and interpretation was iterative to allow adaptations and refinement of the research question, focus group guides, sampling strategy and codes [15]. After each focus group, a debriefing was performed between the moderator (IS) and the observer to reflect on the session. The first author (MS) read and open coded the transcripts. Independently, two researchers from the research group also read and open coded the first two transcripts. Initial codes were then clustered into overarching codes by using axial and selective coding. All stages of coding were discussed and codes were adapted until agreement was reached. Furthermore, memos and a logbook were kept and along with frequent discussions with the research team guided the process of creating understanding of and assigning meaning to the data. The research team consisted of experts with various backgrounds (e. g. doctors, policymakers, professors), with the first author being a health scientist. Eventually, 217 codes resulted in 52 organizing codes grouped into four mechanisms that describe how the HECs strive to enable educational CQI in residency training. Software for qualitative research (MAXQDA) was used to support coding.

## Results

We found that HECs are striving to enable CQI for PGME by (i) creating an organization-wide educational CQI culture, (ii) an organization-wide educational CQI structure and by (iii) collaborating with external stakeholders. Although we did not see any chronological order in these mechanisms, we did identify that HECs were foremost eager to (iv) strategically position themselves in the organization to build the authority to intervene. This claim for power was a prerequisite for HECs to be successful in enabling CQI. All four mechanisms were ongoing and interdependent and HECs needed to balance these to maximize impact on the quality of residency training.

### Mechanism 1: HECs claim a strategic position in the organization

Despite legislation dictating the role of the HEC, we identified that HEC members had to claim a position within teaching hospitals. Respondents mentioned the struggle of prioritizing education in hospitals and of getting a hold as an education committee in the hospital context. An example was provided by HEC 2, stating *‘Until now, it (the value of PGME) was not mentioned in the strategy of the hospital’, *which illustrates PGME is often under prioritized at the hospital level. Therefore, HEC members stressed formalizing their role in hospitals’ statutory documents and negotiating their financial independency as crucial to gaining the right to intervene in residency programs in difficulty. Since hospital boards were felt not to prioritize education and sometimes lacked the will to invest in PGME, respondents identified these boards as the party to negotiate their formal position with. Participants also mentioned that a solid strategic position enhanced the credibility of the HEC, which was especially required to guarantee the success of interventions aimed at residency programs of colleague medical specialists.

The achievements of HECs seemed to vary: some HECs formulated clear policies or obtained their own budgets, others were still working on the contents of such policy or were awaiting approval from the hospital board for their financial independency.

### Mechanism 2: HECs create an organization-wide educational CQI culture

HEC members mentioned an organization-wide educational CQI culture as core to achieving excellent PGME. HECs were said to work on culture amongst, for example, program directors, educators and residents, with the aim to engage them in CQI. HECs enabled awareness about the relevance of qualitatively sound education by continuously addressing strengths and weaknesses of residency programs and their implications, but also by facilitating exchange of best practices between residency programs. The importance of this exchange for PGME was stressed by HEC 1, stating: *‘Things are shared, even when they are not going well. So you can learn from each other. But it also means you can be called upon aspects so you can improve them’. *This quote underlines how sharing practices contributes to improvement of PGME through learning about strengths and weaknesses. To facilitate this exchange, HECs supported a psychologically safe environment to achieve openness, transparency, approachability and ‘speaking-up’. HEC members further expressed the importance of goodwill of the staff in the teaching hospital, to assure willingness to cooperate with the HEC. It was explicitly mentioned that HECs will not start a quality movement on their own and need support from people throughout the organization.

We noted that some HECs have already succeeded in creating an open environment in which speaking-up and transparency had become common practice. These HECs often had a proactive attitude towards CQI and were continuously working towards improvement of PGME. Others had just started to work towards transparency about their CQI policies. These HECs mainly displayed reactive behaviour and intervened only when residency programs had quality issues.

### Mechanism 3: HECs create an organization-wide educational CQI structure

Besides creating a culture, respondents mentioned the need for an educational CQI structure to evaluate and improve PGME organization-wide and to define when and what actions concerning PGME are taken. Within this structure, respondents described the HECs as an overarching body from which CQI efforts for PGME are monitored and coordinated. As a result, HECs were said to initiate the use of systematic approaches, such as PDCA cycles, at the level of individual departments as well as at the organizational level. HMC 6 illustrated this as follows: *‘We are working on (…) a PDCA cycle at the level of the training programs and (…) at the level of the teaching hospital as a whole. We have installed cycles throughout the organization (…) to reach improvement in education’*. Moreover, respondents mentioned the importance of encouraging people to commit to CQI for PGME in order to consolidate quality initiatives. HMC 1 posed: *‘You can make plans, but without the support of clinical teachers and residents those plans are not going to happen. (…) Making sure people actually commit to long-term improvement is difficult’.*

Next, HEC members were found to collaborate with PGME stakeholders throughout the hospital, which supported the translation of HEC policy into action. The need for such a backbone was illustrated by HEC 2, stating: *‘But that whole PDCA cycle, it requires an investment in human resources.. You need to have a structure for that’. *Therefore, respondents indicated that human resources are a necessary condition to enable CQI in PGME. To create these resources, respondents collaborated with the hospital board to acquire funds and to discuss national and international developments in PGME and patient care. Collaboration with both educators and residents was necessary to set up appropriate data collection concerning the quality of residency programs as well as to receive performance feedback from educators and residents themselves. Furthermore, respondents underlined the value of empowering residents in CQI for PGME, since they are the ‘consumers’ of the education. Finally, it was stated that educationalists, along with secretarial staff, often took part in HEC meetings to support and inform the HEC on educational policy.

Some HECs deemed themselves successful in implementing approaches such as PDCA cycles at various levels of the organization, others were mainly working at the level of the residency programs. Some HECs had access to supporting staff and as a result lighter administrative and executive loads, whereas other HECs were practically operating on their own.

### Mechanism 4: HECs work together with external stakeholders

HEC members collaborated with external stakeholders to improve their policy within their own hospital and to strengthen the position of the teaching hospital itself. Respondents mentioned collaborations with other HECs and hospitals from which HECs could organize educational events for residents or educators (e. g. joint trainings). External coaches were involved in training or departmental group discussions about the results of quality evaluations. Such coaches were considered to provide impartiality and anonymity and therefore stimulate speaking-up.

Respondents mentioned that hospitals have the responsibility to guarantee that their residency programs live up to the standards that are set by external bodies and society. HEC 1 illustrated this by stating: *‘If you want a vision on what should be happening in those postgraduate medical training programs you will need a voice from the outside world’. *This quote shows that respondents were of the opinion that education should fit societal demands. However, HECs indicated that priorities set by society are not always acknowledged by educators or residents and, therefore, the HEC has to take the lead in incorporating this voice into their policy.

## Discussion

HECs are striving to enable CQI in PGME by (i) creating an educational CQI culture, (ii) as well as an educational CQI structure and (iii) by collaborating with external stakeholders. These mechanisms are continuously supported by (iv) HECs building a strategic position in the organization. We found these identified mechanisms to strengthen each other in a positive way, potentially creating a virtuous cycle towards high-quality PGME.

### Governance

Many countries are initiating stronger and more holistic governance structures for PGME to improve the quality of education and to keep up with changes in the clinical field [18]. In many leading healthcare systems governance of PGME is set and assessed by external bodies [7, 18, 19]. In Canada, for example, residency programs operate in a university-based organizational structure from which quality is governed per specialty [18, 19]. In many of these systems there is a lack of accountability for teaching hospitals and leaders of these hospitals should be more engaged in PGME and resulting issues [18]. From this perspective, HECs in the Netherlands are uniquely contributing to this issue by creating a central position from which teaching hospitals can be held accountable for the quality of PGME. This centrality might facilitate exchange of resources, enhance interdisciplinary programs and support CQI efforts to guarantee high-quality PGME.

### Implementation

Implementation of CQI for PGME might be challenging, since it might be perceived as burdensome, bureaucratic or as interfering by clinical departments [20–22]. The clinical environment has proven to be rather unreceptive to educational policy when education is considered to be secondary to patient care and medical research [23]. Examining implementation of similar hospital-wide bodies for clinical governance, literature shows the importance of aligning organizational and departmental levels to impact patient outcomes [24]. Such a cooperation was also stressed by our respondents, stating CQI efforts for PGME will not succeed without involvement of departments. Implementing committees such as HECs, shared responsibility of the HEC and departments to achieve high-quality PGME should be addressed. HECs keep the bird’s eye view and intervene when necessary, but medical specialists managing residency training at departments should take on the final responsibility to deliver high-quality education.

### Strategy

Program directors are members of the HECs. These program directors fulfil simultaneously the role of committee member, ergo monitoring peers providing PGME, and of clinical educator, thus being colleagues of their peers providing PGME. This might imply that HEC members are placed in a hierarchical position towards colleague medical specialists. As a result, equivalent relationships between medical specialists may become pressured when members of the HECs execute their right to speak-up and even intervene in residency programs in which fellow specialists are involved. A solid strategic position as HEC then becomes even more important to assure credibility and decisional power.

### Implications for research and practice

With a call for strong educational governance and increased local management of PGME [18], we think that research should focus on the extent to which HEC-like bodies can meet such requirements. To support the potential of HECs, committees in the Netherlands are becoming more important and are assigned a more prominent leadership role in PGME. Moreover, the Dutch Registration Committee for Medical Specialties recently announced its plan for external audits of the functioning of the HECs [25]. Research could investigate whether committees that perform well in these audits are providing better PGME.

For healthcare systems that wish to implement HEC-like bodies to govern PGME, this study informs on mechanisms through which such committees can positively impact PGME. For these healthcare systems, guaranteeing that HEC-like bodies have a strong strategic position through which they can exert power and influence on CQI in PGME is a first important step towards successful HECs.

Furthermore, governance of PGME should commit to maintaining the connection between education and patient care [18]. Especially since residency training takes place in the same context as patient care, we imply that those interested in working towards institutional accountability for PGME can learn from the experience and knowledge assimilated by research into clinical governance. We think collaboration between the two fields might lead to a more integrated system that can benefit education as well as patient care.

This research refrains from investigating effects of HECs’ actions, but respondents addressed the positive effects of CQI on the quality of education (e. g. an increase in resident applications and significant changes in residency programs when compared with previous years). Effects on patient care were mentioned by only one HEC. To decide whether the implementation of HECs actually contributes to the quality of PGME, research into the effects of HECs’ actions is necessary.

### Strengths and limitations

This study was performed in PGME in the Netherlands, so results are bound to the context in which the data were gathered. However, HEC-like bodies implemented in other healthcare systems (e. g. the Graduate Medical Education Committees in the United States) received comparable roles and purposes and have to function in a comparable environment (teaching hospitals). Therefore, we consider the four identified mechanisms to be relevant to HEC-like bodies outside the Netherlands.

Since this research provides a first insight into how HECs are striving to enable CQI in PGME, we only focused on the perspectives of the members of the HECs who seem to be engaged and enthusiastically involved in CQI for PGME. Therefore, we stress that investigating other perspectives, such as from hospital boards, medical specialists and residents, may create additional knowledge about the value of HECs for PGME.

A strength of this study is the iterative and systematic approach we used to analyze the data, thereby refraining from enforcing existing assumptions and models on the data. This approach contributed to the aim of developing an understanding of the value of HECs for the quality of residency training.

## Conclusions

HECs strive to enable CQI for PGME by creating an organization-wide educational CQI culture, structure and by collaborating with external stakeholders. However, HEC members need to claim a strategic position before they are able to bring CQI for PGME forward. HECs seem to increasingly have the ability to undertake supporting actions and are working step by step to contribute to high-quality residency training.
